# Pan-tumor T-lymphocyte detection using deep neural networks: Recommendations for transfer learning in immunohistochemistry

**DOI:** 10.1016/j.jpi.2023.100301

**Published:** 2023-02-27

**Authors:** Frauke Wilm, Christian Ihling, Gábor Méhes, Luigi Terracciano, Chloé Puget, Robert Klopfleisch, Peter Schüffler, Marc Aubreville, Andreas Maier, Thomas Mrowiec, Katharina Breininger

**Affiliations:** a Pattern Recognition Lab, Department of Computer Science, Friedrich-Alexander-Universität Erlangen-Nürnberg, Erlangen, Germany; b Merck Healthcare KGaA, Darmstadt, Germany; c Department Artificial Intelligence in Biomedical Engineering, Friedrich-Alexander-Universität Erlangen-Nürnberg, Erlangen, Germany; d Department of Pathology, University of Debrecen, Debrecen, Hungary; e Research Department Pathology, Universitätsspital Basel, Basel, Switzerland; f Institute of Veterinary Pathology, Freie Universität Berlin, Berlin, Germany; g Institute of General and Surgical Pathology, Technical University of Munich, Munich, Germany; h School of Computation, Information and Technology, Technical University of Munich, Munich, Germany; i Technische Hochschule Ingolstadt, Ingolstadt, Germany

**Keywords:** Tumor-infiltrating lymphocytes, Immuno-oncology, Immunohistochemistry, Deep learning, Transfer learning, Domain adaptation

## Abstract

The success of immuno-oncology treatments promises long-term cancer remission for an increasing number of patients. The response to checkpoint inhibitor drugs has shown a correlation with the presence of immune cells in the tumor and tumor microenvironment. An in-depth understanding of the spatial localization of immune cells is therefore critical for understanding the tumor’s immune landscape and predicting drug response. Computer-aided systems are well suited for efficiently quantifying immune cells in their spatial context. Conventional image analysis approaches are often based on color features and therefore require a high level of manual interaction. More robust image analysis methods based on deep learning are expected to decrease this reliance on human interaction and improve the reproducibility of immune cell scoring. However, these methods require sufficient training data and previous work has reported low robustness of these algorithms when they are tested on out-of-distribution data from different pathology labs or samples from different organs. In this work, we used a new image analysis pipeline to explicitly evaluate the robustness of marker-labeled lymphocyte quantification algorithms depending on the number of training samples before and after being transferred to a new tumor indication. For these experiments, we adapted the RetinaNet architecture for the task of T-lymphocyte detection and employed transfer learning to bridge the domain gap between tumor indications and reduce the annotation costs for unseen domains. On our test set, we achieved human-level performance for almost all tumor indications with an average precision of 0.74 in-domain and 0.72–0.74 cross-domain. From our results, we derive recommendations for model development regarding annotation extent, training sample selection, and label extraction for the development of robust algorithms for immune cell scoring. By extending the task of marker-labeled lymphocyte quantification to a multi-class detection task, the pre-requisite for subsequent analyses, e.g., distinguishing lymphocytes in the tumor stroma from tumor-infiltrating lymphocytes, is met.

## Graphical abstract

**Figure:**
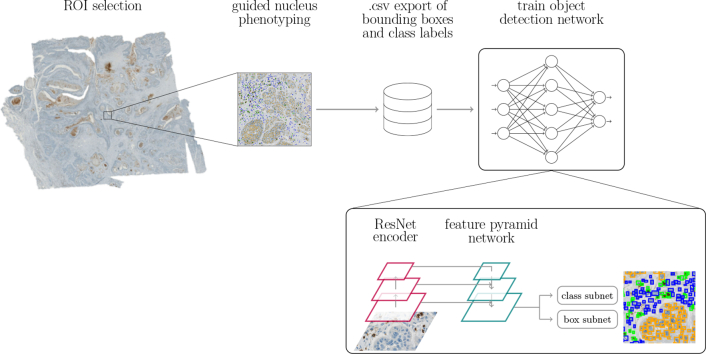

## Introduction

Immuno-oncology is a research field that focuses on leveraging the cancer-immune cell interactions for therapy by, e.g., activating or strengthening the immune response. The success of these treatments varies significantly across patients and recent studies have shown that treatment success is correlated with the presence of tumor-infiltrating lymphocytes (TILs).^1^ Therefore, the detection and quantification of T-lymphocytes have moved into the focus of ongoing research. Specific immunohistochemistry (IHC) stainings can simplify T-lymphocyte quantification. These stainings use target-specific antibodies to bind color-producing enzymes to the objects of interest and the colored precipitates (e.g., 3,3’-diaminobenzidine) are then assessed using light microscopy.^2^ The visual estimation of T-lymphocytes, however, is prone to high inter-observer variability and the manual quantification can be very cumbersome and time-consuming.^1^

The introduction of slide scanners into pathology workflows has enabled the digitization of histological samples and thereby the use of computer-aided diagnosis (CAD) tools for image processing and analysis. These CAD systems can help to speed-up workflows and increase the reproducibility of image analysis results. However, most of these systems are based on traditional color appearance parameters, e.g., hue and contrast, and require manual threshold optimization for each slide.^3^ Color appearance can vary significantly across samples due to sample age, artifacts, sample composition (e.g., presence of metal ions), or varying staining protocols of different pathology labs. Therefore, more robust image analysis methods that allow for a higher level of automation by also taking into account texture features are highly desirable.

Convolutional neural networks (CNNs) have become increasingly popular for a wide range of image processing tasks and have been successfully applied for the task of lymphocyte quantification.^4^, ^5^, ^6^, ^7^, ^8^, ^9^ In contrast to traditional machine learning-based algorithms, CNNs do not require hand-crafted features for training. Through trainable parameters, they can learn the extraction of task-relevant features, thereby become end-to-end trainable, and reduce the bias introduced by manual feature selection. When using CNNs for the quantification and localization of individual lymphocytes, this can be posed as an object-detection task, where the network is trained with labeled bounding boxes around each cell of interest. Evangeline *et al* used the Faster RCNN object detection architecture to detect lymphocytes on whole slide images (WSIs) from 3 organs stained with 2 IHC markers.^7^ Van Rijthoven *et al* deployed the YOLOv2 architecture on breast, colon, and prostate cancer samples stained with IHC.^8^ In a subsequent study,^9^ the authors deployed this approach on an extended dataset of 83 WSIs from 3 organs, 9 medical centers, and 2 staining types. Previous work in histopathology has shown that algorithmic performance can considerably degrade when testing the models on out-of-distribution data,^10^ which can be compensated for by using domain adversarial training^11^ or fine-tuning.^12^ However, previous studies on automated T-lymphocyte detection did not evaluate transfer learning techniques for this task, even though domain shifts were present in the datasets and cross-domain performance degradation was observed for some of these works.^9^

In this work, we explicitly study the robustness of an object detection algorithm for the task of T-lymphocyte detection under various influence factors. We train the algorithm with a varying number of IHC images and thereby evaluate the algorithm performance dependent on the number and diversity of samples seen during training. Furthermore, we extend the task of IHC-stained T-lymphocyte detection to a multi-class problem by including tumor cells and remaining cells in the tumor stroma. We then test the algorithm on images from different tumor indications and study the robustness of cell detection and classification under this domain shift. Due to a performance drop across tumor indications, we employ transfer learning to increase the algorithm’s robustness and recover performance. Based on these experiments, we provide recommendations for the development of robust algorithms for T-lymphocyte quantification on IHC images, especially for applications where limited data is available or domain shifts are introduced.

## Material and methods

In the course of this study, a total of 92 procured, anonymized, commercially acquired human tumor samples from 4 tumor indications were used:

(1.) 32 head and neck squamous cell carcinoma (HNSCC) samples;
(2.) 20 non-small cell lung cancer (NSCLC) samples;
(3.) 20 triple-negative breast cancer (TNBC) samples;
(4.) 20 gastric cancer (GC) samples.

The procured tissue samples were obtained from 3 providers (Asterand, Cureline, and Tristar), which all guarantee institutional review board (IRB) approval. Table A.1 provides a detailed slide-level overview of the samples used, including a diagnosis and the tumor-node-metastasis (TNM) staging,^13^ where available. All samples were fixed in formalin, embedded in paraffin, and IHC stained for cluster of differentiation 3 (CD3, antibody clone SP7). CD3 is a protein complex with a specificity for T-lymphocytes. For each sample, the IHC staining underwent manual quality control to guarantee uniform staining results and detect staining artifacts. The samples were digitized at a resolution of 0.23 μm/px (40× objective lens) using the NanoZoomer 2.0-HT scanning system (Hamamatsu, Japan). All samples were prepared and digitized at the same laboratory (Merck Healthcare KGaA). For model development, 5 validation and 5 test slides were randomly selected per tumor indication. A detailed slide-level split can be obtained from Table A.1. On each WSI, a squared region of interest (ROI) sized approximately 2 mm^2^ (∼2150 × 2150 pixels) was randomly selected from regions containing both tumor and tumor stroma, as well as CD3^+^ stained cells. We provide public access to all selected ROIs on Zenodo (https://doi.org/10.5281/zenodo.7500843), licensed under a creative commons attribution-non-commercial 4.0 international license.

### Data annotation

**Fig. 1:**
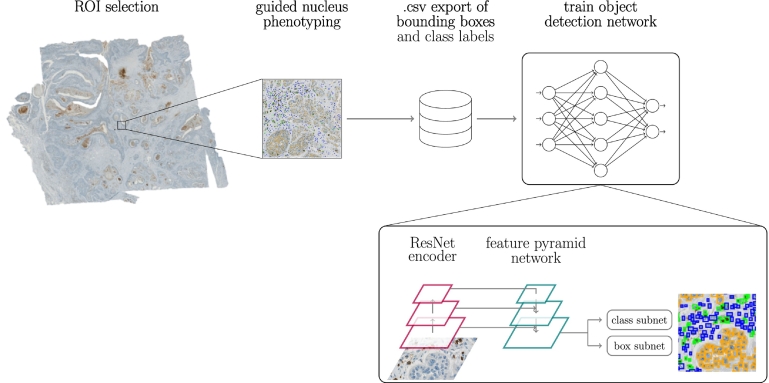
Annotation and training pipeline. ROIs sized 2 mm^2^ are annotated using commercially available image analysis software.

In the selected ROIs, all CD3^+^ and tumor cells were annotated individually. All remaining stromal cells, not stained positive for CD3, were combined into a third class, which we will refer to as “non-specified cells”. These annotations were produced in a semi-automatic fashion using commercially available image analysis software (HALO®, Indica Labs, USA). This software uses manually selected cell prototypes to train an underlying algorithm for the task of cell segmentation and phenotyping. To ensure a high annotation quality, we optimized this algorithm for each selected ROI individually. We exported the bounding box vertices and class label of all detected cells as .csv file, which we then used as an annotation database to train an algorithm for automatic cell detection and classification. With this annotation pipeline, visualized in Fig. 1, a high number of cell annotations could be generated in a comparably short time frame. Nevertheless, this semi-automatic pipeline might miss individual cells or introduce false-positive annotations. Therefore, we compared the semi-automatically created annotations to manual expert labeling on our test set. For these manual annotations, we used a private instance of the online annotation server EXACT.^14^ Using EXACT, 3 pathologists independently annotated each of the 5 test ROIs per tumor indication for the 3 cell classes. To limit annotation overhead, we asked the pathologists to label the approximate cell center with one-click annotations and defined the cell’s bounding box using a width of 25 pixels (the radius of an average cell) around the cell center. A cell should be identified through either the blue counter stain of the nucleus or the cytoplasmic IHC staining of T-lymphocytes. All annotations can be accessed on Zenodo (https://doi.org/10.5281/zenodo.7500843).

### Metrics for inter-annotator agreement

Previous studies have shown that many object-level tasks in pathology, like mitotic figure detection^15^ or cell quantification on cytology samples,^16^ can be affected by a high level of inter-annotator variability, which can influence the performance of algorithms trained for these object-level tasks.^15^ To estimate the inter-annotator variability for the task of T-lymphocyte detection, we evaluated the agreement of 3 pathologists on our 20 test ROIs. For this, we have used adapted versions of Cohen’s kappa.^17^ For 2 raters A and B, Cohen’s kappa is defined as:

(1) $$\kappa_{\mathrm{AB}}=\frac{P_{o}-P_{e}}{1-P_{e}},$$

where *P*_*o*_ is the observed percent agreement, i.e. the proportion of cells for which 2 pathologists assigned the same label, and *P*_*e*_ is the expected percent agreement, i.e., the proportion of cells on which the pathologists agreed simply by chance. Given a 3-class problem with class labels *c*_*i*∈{1,2,3}_, *P*_*o*_ and *P*_*e*_ can be computed from the confusion matrix as follows:

(2) $$\begin{array}{llllll} & & & \text{Rater B} & & \\ & & c_{1} & c_{2} & c_{3} & \Sigma \\ & c_{1} & n_{11} & n_{12} & n_{13} & n_{1\cdot }\\ \text{Rater A} & c_{2} & n_{21} & n_{22} & n_{23} & n_{2\cdot }\\ & c_{3} & n_{31} & n_{32} & n_{33} & n_{3\cdot }\\ & \Sigma & n_{\cdot 1} & n_{\cdot 2} & n_{\cdot 3} & N \end{array}$$

(3) $$\begin{array}{l} P_{o}=\frac{1}{N}\sum_{i=1}^{3}n_{\mathrm{ii}}\\ P_{e}=\frac{1}{N^{2}}\sum_{i=1}^{3}n_{i\cdot }\cdot n_{\cdot i} \end{array}$$

Landis and Koch have defined the following intervals,^18^ which can be used to interpret kappa scores:

$$\begin{array}{rr} \kappa <0: & \text{poor agreement}\\ 0<\kappa \leq 0.2: & \text{slight agreement}\\ 0.2<\kappa \leq 0.4: & \text{fair agreement}\\ 0.4<\kappa \leq 0.6: & \text{moderate agreement}\\ 0.6<\kappa \leq 0.8: & \text{substantial agreement}\\ 0.8<\kappa \leq 1.0: & \text{almost perfect agreement}. \end{array}$$

Previous work has shown that object detection tasks in histopathology are prone to omission of cells.^16^^,^^19^ De Raadt *et al* presented 3 variants of Cohen’s kappa for missing data: The Regular Category kappa, the Listwise Deletion kappa, and Gwet’s kappa.^20^ The Regular Category kappa considers missed cells a separate class and thereby extends a 3-class problem to a 4-class problem, whilst keeping the computation of kappa unchanged. The Listwise Deletion kappa maintains the definition as a 3-class problem and limits the computation of kappa to cells that have been labeled by both raters. Gwet’s kappa excludes cells that have only been labeled by 1 rater from the observed percent agreement *P*_*o*_ but includes them in the computation of the expected percent agreement *P*_*e*_. With t, being the class of non-labeled cells, the confusion matrix in Equation (2) can be extended to:

(4) $$\begin{array}{ccccccc} & & & \text{Rater B} & & \\ & & c_{1} & c_{2} & c_{3} & c_{t} & \Sigma \\ & c_{1} & n_{11} & n_{12} & n_{13} & n_{1t} & n_{1\cdot }\\ \text{Rater A} & c_{2} & n_{21} & n_{22} & n_{23} & n_{2t} & n_{2\cdot }\\ & c_{3} & n_{31} & n_{32} & n_{33} & n_{3t} & n_{3\cdot }\\ & c_{t} & n_{t1} & n_{t2} & n_{t3} & n_{\mathrm{tt}} & n_{t\cdot }\\ & \Sigma & n_{\cdot 1} & n_{\cdot 2} & n_{\cdot 3} & n_{\cdot t} & N \end{array}$$

The components of the 3 kappa variants are then calculated as follows:

**Regular Category:**

(5) $$\begin{array}{l} P_{o}=\frac{1}{N}\left(\left(\sum_{i=1}^{3}n_{\mathrm{ii}}\right)+n_{\mathrm{tt}}\right)\\ P_{e}=\frac{1}{N^{2}}\left(\left(\sum_{i=1}^{3}n_{i\cdot }\cdot n_{\cdot i}\right)+n_{t\cdot }\cdot n_{\cdot t}\right) \end{array}$$

**Listwise Deletion:**

(6) $$\begin{array}{l} P_{o}=\frac{\sum_{i=1}^{3}n_{\mathrm{ii}}}{\sum_{i=1}^{3}\sum_{j=1}^{3}n_{\mathrm{ij}}}\\ P_{e}=\frac{\sum_{i=1}^{3}\left(n_{i\cdot }-n_{\mathrm{it}}\right)\left(n_{\cdot i}-n_{\mathrm{ti}}\right)}{{\left(\sum_{i=1}^{3}\sum_{j=1}^{3}n_{\mathrm{ij}}\right)}^{2}} \end{array}$$

**Gwet:**

(7) $$\begin{array}{l} P_{o}=\frac{\sum_{i=1}^{3}n_{\mathrm{ii}}}{\sum_{i=1}^{3}\sum_{j=1}^{3}n_{\mathrm{ij}}}\\ P_{e}=\frac{\sum_{i=1}^{3}n_{i\cdot }\cdot n_{\cdot i}}{\left(N-n_{t\cdot }\right)\cdot \left(N-n_{\cdot t}\right)} \end{array}$$

Using these adapted versions of kappa, the 2 causes for inter-observer variability - cell omission and label disagreement - can better be separated and a pathologist consensus can better be defined.

### Algorithm robustness experiments

Using the semi-automatically annotated images of our training dataset, we trained a neural network for the task of cell detection and classification into tumor, CD3^+^, and non-specified cells. For all our experiments we used a customized RetinaNet^21^ architecture adapted for cell detection on microscopic samples.^16^ This architecture is visualized in Fig. 1. RetinaNet is composed of an encoding branch, for which we used a ResNet18^22^ backbone, and a feature pyramid network that combines features from multiple encoder levels. From the combined features, the network then infers object bounding boxes and classifies the object within. We conducted 3 experiments to evaluate the algorithm’s robustness under different influence factors. We first performed a WSI ablation study, where we included an increasing number of images in the training subset for the algorithm and evaluated the influence of this slide variability seen during training on the predictive power of the algorithm on unseen test images. Afterward, we compared the algorithmic performance on images from the same image domain that the model was trained on, i.e., the source domain, to unseen target domains. Finally, we used fine-tuning to bridge the domain gap between source and target domain images. We will elaborate on these experiments in the following 3 subsections.

#### WSI ablation study

For this experiment, we only used the HNSCC dataset of 32 WSIs. To evaluate the algorithm robustness dependent on the number of slides used for training, we conducted a WSI ablation study. For this, we trained the RetinaNet with an increasing number (1–10, 15, 22) of WSIs and tested the algorithm performance on our 5 test ROIs. The training slides were hereby randomly selected from the pool of 22 training WSIs and each experiment was repeated 5 times with a different random selection. For each experiment, we used the same set of 5 validation WSIs to monitor training, prevent overfitting, and guide the model selection process. These validation slides were randomly selected from the complete dataset at the beginning of the study. In the remainder of the text, we will refer to the models of the WSI ablation study as RetinaNet_*n*_, with *n* indicating the number of HNSCC WSIs used for training.

#### Deployment of source model on target domains

To evaluate the model robustness across different tumor indications, we deployed all models from the WSI ablation study on 5 test WSIs each of NSCLC, TNBC, and GC without adaptations to this new domain. In the following, we will refer to HNSCC as “source domain” and the remaining tumor indications as “target domains”. For each target domain, we trained an additional benchmark model from scratch on 10 training and 5 validation WSIs to evaluate whether the source-domain model can reach target-domain performance. In the following, we will refer to these benchmarks as RetinaNet_*NSCLC*_, RetinaNet_*TNBC*_, and RetinaNet_*GC*_. To compensate for statistical effects, we also repeated the benchmark training 5 times and averaged the performance results.

#### Model fine-tuning on target domains

To overcome a potential domain shift between the different tumor indications, we evaluated how fine-tuning on a few target domain samples influences the model performance on the target domain. We further investigated how the number of slides used to train the initial source model influenced this fine-tuning. To maintain good performance on the source domain, the fine-tuning dataset was composed of the *n* HNSCC WSIs the model was initially trained on and 1 additional WSI of the respective target domain. This additional training WSI was chosen at random but was kept the same for each of the fine-tuned models. The validation set, used for model selection and hyperparameter optimization, was kept unchanged as 5 HNSCC WSIs. In the remainder of the text, we will refer to these fine-tuned models as RetinaNet_*n*,*T*_, where *n* indicates the number of HNSCC WSIs initially used for training and *T* the tumor indication the model was fine-tuned on.

### Training hyperparameters

For all experiments, we trained the network on image patches sized 256 × 256 pixels at the original resolution of $0.23\frac{\mu m}{\mathrm{px}}$, using a batch size of 16. For each training epoch, we followed a random sampling strategy to select 5000 patches from the training ROIs and 800 patches from the validation ROIs. The models trained from scratch (ablation and benchmark models) were initialized with ImageNet^23^ weights, which was proven to be advantageous for microscopy data compared to random initialization.^24^^,^^25^ Furthermore, previous work has shown that networks fine-tuned on histopathology data especially differ in their feature representation of deeper layers whilst earlier layers show similar activation patterns to networks trained on ImageNet.^24^ Therefore, similar to Tajbakhsh *et al,*^26^ we followed a 2-staged fine-tuning scheme: During the first stage, we only trained the prediction heads for 5 epochs while freezing the encoder and feature pyramid pooling network weights. For this stage, we used a discriminative^27^ learning rate in an interval of [5 × 10^−5^, 5 × 10^−4^]. During the second stage, we trained the whole network with a discriminative learning rate in an interval of [5 × 10^−5^, 10^−4^] for 50 epochs. The intervals for the discriminative learning rate were estimated using the learning rate finder of fastai.^28^ When fine-tuning the models on a target domain, we again froze the encoder and feature pyramid pooling network weights and only trained the prediction heads. For all models, the loss was computed as the sum of the bounding box regression loss, calculated as smooth L1 loss, and the instance classification loss using the focal loss function.^21^ To avoid overfitting, model selection was guided by the highest performance on the validation set, assessed by monitoring the mean average precision (mAP) after each epoch.

### Performance evaluation

After patch-wise model training, we applied the trained models to the 2 mm^2^-sized test ROIs by extracting patches with a 128-pixel overlap and applying non-maximum suppression (NMS) to remove duplicate detections. Afterward, detection results were evaluated against the annotations. We used the following approach to automatically find correspondences between detected and annotated cells: From the set of annotations *A* and the set of detections *D*, a distance matrix *M* was generated, with *m*_*ij*_ being the Euclidean distance between element *a*_*i*_ ∈ *A* and *d*_*j*_ ∈ *D*. A unique pairwise assignment with the lowest overall cost was computed using the Hungarian Algorithm.^29^ A pair-wise assignment was taken into consideration if the annotated cell centroid and the centroid of a predicted bounding box were within 25 pixels of each other, i.e., the radius of an average cell. Otherwise, the detection was counted as false, and the annotation as missed. Using these cell correspondences a confusion matrix could be generated, summarizing true-positive (TP), false-positive (FP), and false-negative (FN) predictions. Given the example of tumor cells, TP predictions were defined as all tumor cells detected and classified as such. FN predictions were defined as all cells labeled as tumor cells but predicted as another cell type or not detected at all. FP predictions included all cells detected and classified as tumor cells but labeled as non-specified or CD3^+^ cells or not annotated at all.

Commonly, object detection algorithms are evaluated using the average precision (AP). For each detection, the algorithm outputs a score in the range of [0, 1]. Thresholding these predictions with a detection threshold *σ*_*det*_ results in varying precision–recall pairs when evaluating the detections against the ground-truth annotations. Using these precision–recall pairs, the AP can be computed as the weighted sum of precisions, where each precision value is weighted with the increase in recall between 2 *σ*_*det*_. If *σ*_*det*_ is chosen with a step size of 0.1, the AP can be computed from 11 precision–recall pairs according to:

(8) $$\mathrm{AP}=\sum_{k=1}^{10}\left(\text{Recall}\left(k+1\right)-\text{Recall}\left(k\right)\right)\cdot \text{Precision}\left(k\right).$$

## Results

### Inter-annotator agreement

**Fig. 2:**
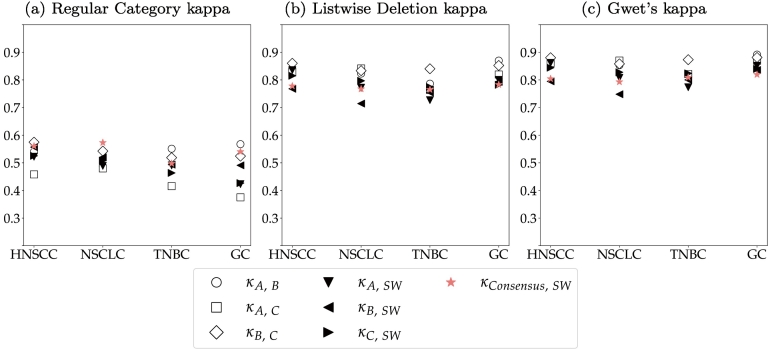
Kappa variants per tumor indication. Annotators A, B, and C are compared to each other (hollow symbols) and to semi-automatic annotations (filled symbols) using the image analysis software (SW). The pathologist consensus is also compared to the software annotations (star).

Fig. 2 visualizes the 3 kappa variants for each unique pair of human raters (hollow symbols) and each rater compared to the semi-automatic labels generated with the image analysis software (filled symbols). These were computed from all annotations on the test ROIs. The large difference between the Regular Category kappa and the other 2 kappa definitions indicates that disagreement between raters was mostly caused by cells that were missed by one of the raters. This especially influenced the agreement of rater A and C, whose Regular Category kappa was the lowest across all tumor indications. Rater B and the semi-automatic annotations were more consistent, which is highlighted by a higher Regular Category kappa for these 2 raters. These different annotation styles also become apparent when comparing the total number of test annotations of all raters. Whilst raters A and C annotated 23 188 and 22 170 cells, rater B and the software provided 28 229 and 29 298 labels. The visualizations of the Listwise Deletion and Gwet’s kappa show that the raters almost perfectly agreed on the cells that they assigned a label for, regardless of the tumor indication. On average, the agreement between the human raters was slightly higher than to the semi-automatic annotations generated with the image analysis software. This difference, however, can be considered marginal and still resulted in substantial to almost perfect agreement. When taking into account the limited availability of human experts and the laboriousness of single-cell annotations (approx. 30 min per ROI), these results support the validity of semi-automatically generating training labels.

When defining a pathologist consensus, a strategy that takes the possibility of incomplete annotations into account has to be found. Under the assumption that pathologists rarely hallucinate cells, we decided to include all cells annotated by the 3 pathologists in the consensus. When more than one pathologist labeled a given cell, the class label was inferred using majority voting. In the case of a draw, i.e., a cell was only annotated by 2 pathologists who assigned a different label, the cell obtained a separate label, which we will refer to as “diverse” in the following. For the complete set of 20 test ROIs, this affected 8.21±2.11 % (*μ* ± *σ*) cells. The consensus annotations were used to evaluate model performance on the test ROIs. Cells labeled as diverse were excluded from the evaluations.

**Fig. 3:**
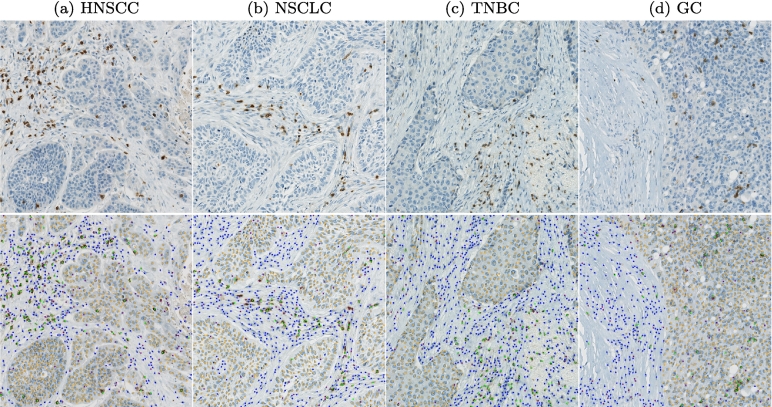
Examples for consensus annotations (orange: tumor cells, green: CD3^+^ cells, blue: non-specified cells, purple: cells without agreement). HNSCC: head and neck squamous cell carcinoma, NSCLC: non-small cell lung cancer, TNBC: triple-negative breast cancer, GC: gastric cancer.

Fig. 3 shows an exemplary test ROI for each tumor indication with the original patch above and the consensus labels below. The examples visualize two main sources of disagreement: The HNSCC sample in Fig. 3a contains large cells that are located in the transition of tumor and tumor stroma and can therefore be interpreted both as tumor and non-specified cells. The TNBC sample in Fig. 3c shows lightly stained cells where differentiation of non-specified and CD3^+^ cells might be difficult for the human annotator.

### WSI ablation and model deployment on target domains

**Fig. 4:**
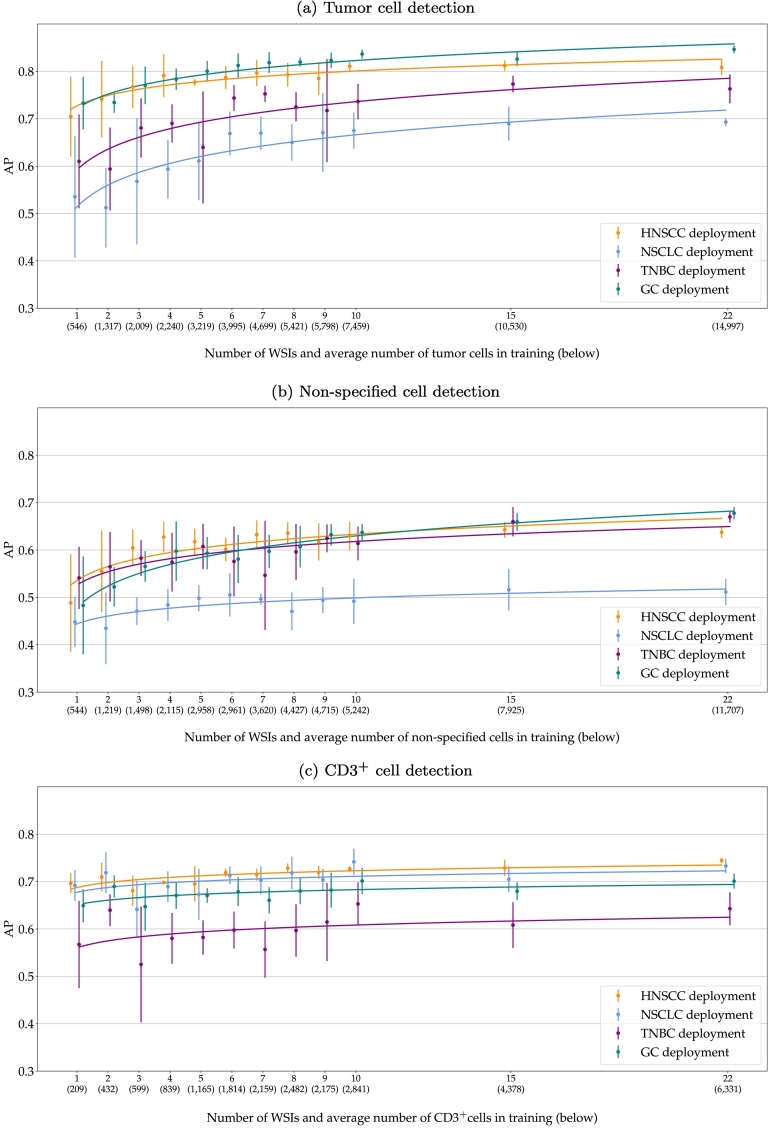
Average precision (AP) when deploying the models on test data from the source domain (HNSCC) and unseen target domains. The models were trained on field of views from an increasing number of whole slide images. The error bars visualize the standard deviation of the 5 repetitions and the curve fits a logistic regression. HNSCC: head and neck squamous cell carcinoma, NSCLC: non-small cell lung cancer, TNBC: triple-negative breast cancer, GC: gastric cancer.

Fig. 4 plots the AP for detecting tumor cells (Fig. 4a), non-specified cells (Fig. 4b), and CD3^+^ cells (Fig. 4c), when training the RetinaNet with an increasing number of slides. The x-axis shows the number of ROIs used for training and the average number of cells present on these ROIs. The bar plots are centered on the mean performance of the 5 training repetitions with the error bars indicating the standard deviation. The curve fits a logistic regression of the AP scores. The tumor cell detection (Fig. 4a) generally improved with more training slides. The regression curves show that the AP increased until about 9 WSIs and then reached a plateau where changes in performance did not exceed the test variance. However, with a higher number of training slides, the model gained robustness, indicated by a much lower variance of the 5 model repetitions for RetinaNet_*22*_. This increase in performance and robustness could also be observed when deploying the models to unseen tumor indications. However, the mean performance and standard deviation highly varied across types. For GC, the source-domain model showed a similar performance as compared to HNSCC. For TNBC, the tumor cell AP was on average 10% lower for RetinaNet_*1*_, which could be recovered to roughly 5% for RetinaNet_*22*_. For NSCLC, RetinaNet_*1*_ performed worst with an AP more than 15% lower compared to the source domain. This could be recovered to 10% for RetinaNet_*22*_. Overall, RetinaNet_*22*_ showed an increased robustness for all tumor indications with a considerably lower model variance across training repetitions.

Compared to the tumor cell detection, the plot of the non-specified cell detection (Fig. 4b) shows a smaller increase in performance with more WSIs used for training. For HNSCC, TNBC, and GC, the cell detection shows similar AP scores across all models with a slightly lower robustness for TNBC, indicated by a higher variance across model repetitions. Again, NSCLC shows the lowest AP values, indicating that a differentiation of tumor and non-specified cells was more challenging for this tumor indication.

The plot of the CD3^+^ cell detection performance in Fig. 4c does not show a large increase in performance when training the algorithm with a higher number of slides in the source domain. RetinaNet_*1*_ already scored an AP of 0.70 on the source domain and AP scores in the range of 0.57 (TNBC)–0.69 (NSCLC) on the target domain. Looking at the average number of cells used for training the model, however, considerably fewer CD3^+^ cells were seen during training, compared to tumor and non-specified cells. For RetinaNet_*22*_, the CD3^+^ classification performance slightly increased to an average of 0.74 on the source domain and a range of 0.64 (TNBC)–0.73 (NSCLC) on the target domains.

**Fig. 5:**
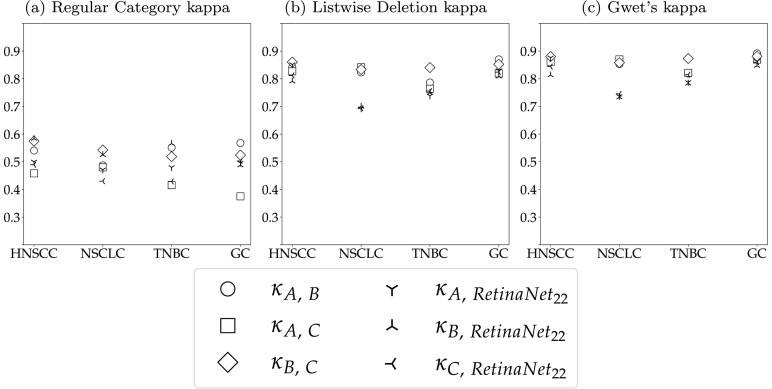
Kappa variants per tumor indication. Annotators A, B, and C are compared to each other (hollow symbols) and to the detection results of RetinaNet_*22*_ (asterisks).

Fig. 5 compares the inter-annotator agreement to the agreement with the detections of the RetinaNet_*22*_ models. For this, we performed inference with an ensemble of all 5 trained models and used NMS to remove duplicates. The visualization shows that the model reached human-level performance for all tumor indications except for NSCLC.

### Qualitative results

**Fig. 6:**
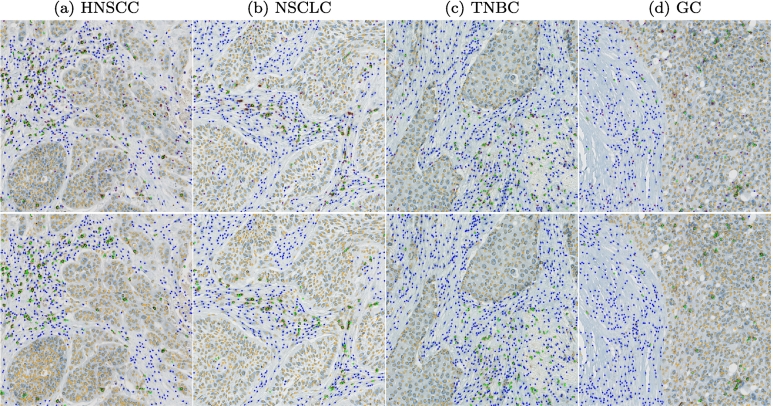
Comparison of consensus annotations in upper row vs. network predictions in the bottom row (orange: tumor cells, green: CD3^+^ cells, blue: non-specified cells, purple: cells without agreement). HNSCC: head and neck squamous cell carcinoma, NSCLC: non-small cell lung cancer, TNBC: triple-negative breast cancer, GC: gastric cancer.

Fig. 6 visualizes the average detection performance of the RetinaNet_*22*_ models on exemplary test ROIs. The upper row shows the consensus annotations and the lower row the network predictions. These visual results underline the strong cell detection performance with few false-positive or false-negative detections. Regarding classification performance, the visual examples show that the differentiation between tumor and non-specified cells was especially difficult at tumor margins, where a majority of the misclassifications could be located.

**Fig. 7:**
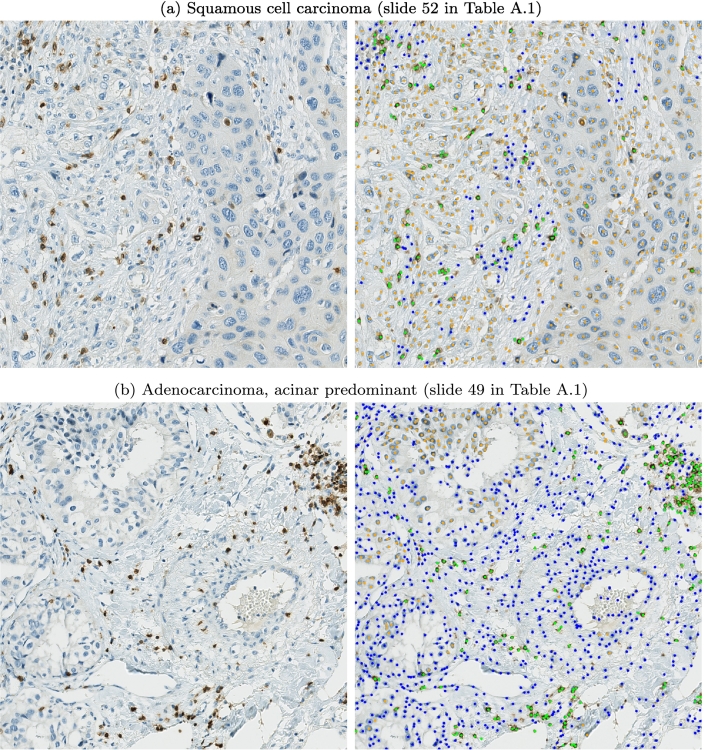
Examples were the models faced difficulties to differentiate tumor and non-tumor cells (orange: tumor cells, green: CD3^+^ cells, blue: non-specified cells).

The slide-level details in Table A.1 show a high diversity of histologic subtypes within our dataset, especially for lung specimens. To evaluate whether the difficulties in tumor/non-tumor cell differentiation were subtype-specific, we assessed the NSCLC predictions in more detail. Fig. 7 visualizes 2 examples where the algorithm faced the most difficulties. The example in Fig. 7a shows a sample where a high amount of cells in the tumor stroma were falsely classified as tumor cells. These cells in the tumor stroma feature an atypically broad cytoplasm that makes differentiation from the larger tumor cells more difficult. Fig. 7b visualizes an adenocarcinoma sample, where a high amount of tumor cells located in the lower left were falsely predicted as non-specified cells. This example generally shows less dense tumor clusters than the examples in Fig. 6, which also makes a visual differentiation of tumor and non-tumor cells difficult. Overall, we observed that tumors with diffuse growth patterns were more challenging for the algorithm. However, we did not observe distinct subtype-specific differences in our dataset.

### Model fine-tuning on target domains

**Fig. 8:**
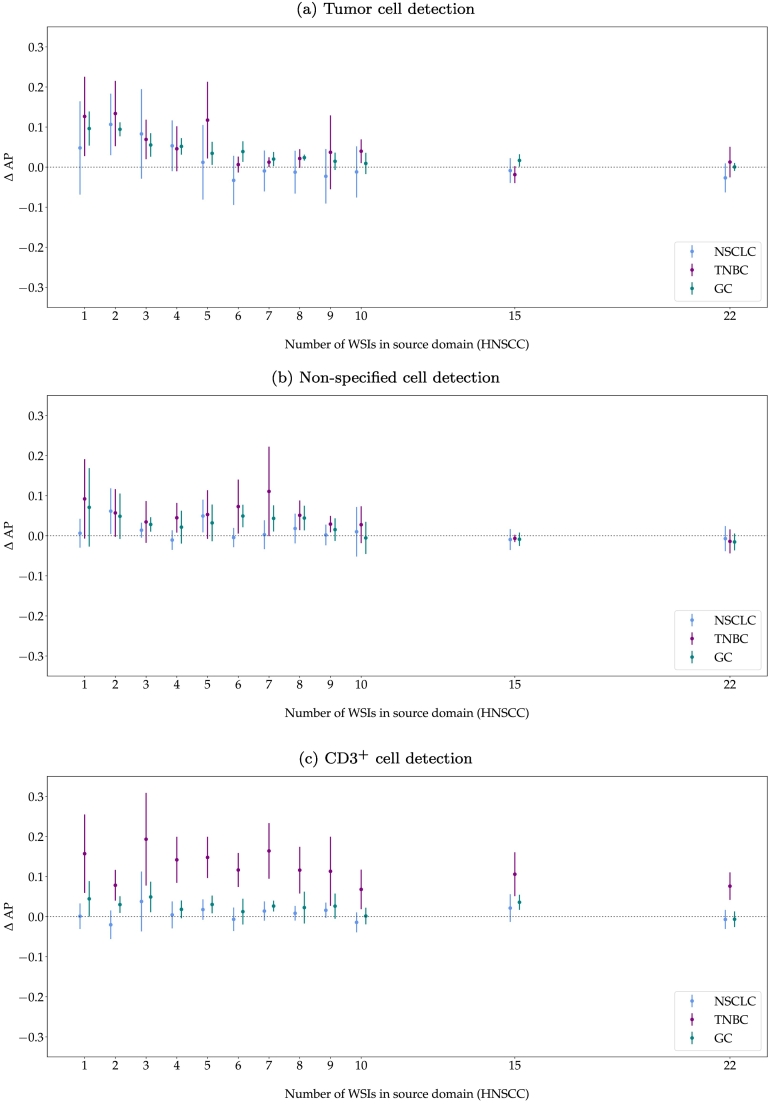
Difference in average precision (AP) for model deployment vs. fine-tuning with 1 target domain image. The error bars visualize the minimum to maximum range of the performance difference. HNSCC: head and neck squamous cell carcinoma, NSCLC: non-small cell lung cancer, TNBC: triple-negative breast cancer, GC: gastric cancer.

Fig. 8 shows the improvement of AP when fine-tuning the models on 1 additional WSI from the respective target domain. Generally, fine-tuning increased the tumor cell detection performance for all target domains, especially for models trained with a low number of WSIs in the source domain or models with low robustness, indicated by a high variance across model repetitions. Whilst fine-tuning improved the performance of most of the models trained with fewer slides in the source domain, RetinaNet_*22*_ did not clearly benefit from fine-tuning on the target domains, indicated by a similar or even slightly worse AP.

**Fig. 9:**
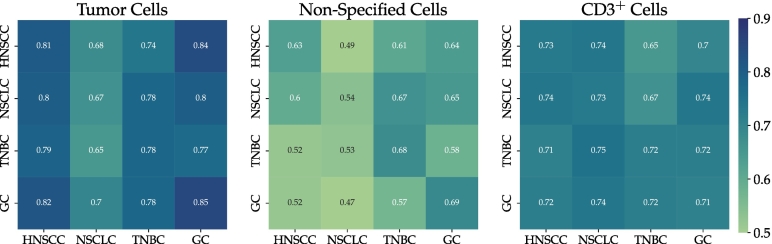
Average performance of benchmark models when being tested on all tumor indications. Matrix entry *m*_*i*,*j*_ is the average precision (AP) when training on the indication in row i and testing on the indication in column j. Diagonal elements indicate in-domain performance, whereas off-diagonal elements represent cross-domain performance. HNSCC: head and neck squamous cell carcinoma, NSCLC: non-small cell lung cancer, TNBC: triple-negative breast cancer, GC: gastric cancer.

Table 1 summarizes the mean AP for models trained with n = 1, 5, 10, and 22 WSIs in the source domain and of the fine-tuned models on their respective target domain *T*. This representation again highlights the improved detection performance when training the model with more WSIs in the source domain but also the smaller benefit of fine-tuning for the models initially trained with more source-domain slides. The highest AP per cell class and per tumor indication (highlighted in bold) was either scored by a model trained with a high number of HNSCC slides or by a fine-tuned model. The last row of Table 1 summarizes the mean performance of the benchmark models trained from scratch on 10 annotated WSIs of the target domains (mean of 5 repetitions). These benchmark models provide a comparison of how good the performance could get if a sufficiently high number of annotated samples were available in the target domain. To limit additional annotation effort and based on the results of the WSI ablation experiments, a selection of 10 target WSIs was used for training the benchmark models. The results show that the best-performing models from the previous experiments reached the performance of the respective benchmark models despite only being trained on the source domain or only fine-tuned with 1 WSI in the target domain. Nevertheless, the benchmark performance also highlights the differences in performance for the 4 tumor indications, especially for the differentiation of tumor and non-specified cells. Fig. 9 shows the average in-domain and cross-domain performance of our benchmark models. This visualization highlights the negligible impact of indication-specific domain shifts when training the model with a sufficiently high number of source domain WSIs, indicated by a similar classification performance within one column. However, the visualization also underlines the challenges posed by the NSCLC dataset as the NSCLC benchmark model scores a comparably low AP across all tumor indications and cell types.

**Table 1:** Average precision for deployment vs. fine-tuning for n = 1, 5, 10, 15, and 22 training whole slide images (mean of 5 training repetitions). The highest average precision per cell class and per tumor indication is highlighted in bold. HNSCC: head and neck squamous cell carcinoma, NSCLC: non-small cell lung cancer, TNBC: triple-negative breast cancer, GC: gastric cancer.

	Tumor cells				Non-specified cells				CD3^+^ cells			
	HNSCC	NSCLC	TNBC	GC	HNSCC	NSCLC	TNBC	GC	HNSCC	NSCLC	TNBC	GC
RetinaNet_*1*_	0.70	0.54	0.61	0.73	0.49	0.45	0.54	0.48	0.70	0.69	0.57	0.65
RetinaNet_*1*,*T*_		0.58	0.74	0.83		0.45	0.63	0.55		0.69	0.72	0.69
RetinaNet_*5*_	0.78	0.61	0.64	0.80	0.62	0.50	0.61	0.59	0.70	0.67	0.58	0.67
RetinaNet_*5*,*T*_		0.62	0.76	0.83		0.55	0.66	0.63		0.69	**0.73**	0.70
RetinaNet_*10*_	**0.81**	0.68	0.74	0.84	0.63	0.49	0.61	0.64	0.73	**0.74**	0.65	0.70
RetinaNet_*10*,*T*_		0.66	**0.78**	**0.85**		0.50	0.64	0.63		0.73	0.72	0.70
RetinaNet_*15*_	**0.81**	**0.69**	0.77	0.83	**0.64**	0.52	0.66	0.66	0.73	0.70	0.61	0.68
RetinaNet_*15*,*T*_		0.68	0.75	0.84		0.51	0.65	0.65		0.73	0.71	**0.72**
RetinaNet_*22*_	**0.81**	**0.69**	0.76	**0.85**	**0.64**	**0.57**	0.70	0.68	**0.74**	0.73	0.64	0.70
RetinaNet_*22*,*T*_		0.67	**0.78**	**0.85**		0.50	0.66	0.66		0.73	0.72	0.69
Benchmark_*10*,*T*_		0.67	**0.78**	**0.85**		0.54	**0.68**	**0.69**		0.73	0.72	0.71

## Discussion

The results of the WSI ablation study show that a higher number of WSIs during model training overall helped to distinguish tumor from non-specified cells as the AP continuously increased for both of these classes. The CD3^+^ cell detection, however, benefited less from a higher number of training images, indicating that the detection of the underlying IHC staining is a much easier task for the network to learn than distinguishing cells from each other based on their morphology. Nevertheless, also tumor and non-specified cell detection reached a plateau in performance at around 9 WSIs. Thus, the marginal increase in performance does not necessarily justify the increased annotation time (approx. 30 min per ROI). The models trained with a higher number of slides are likely to meet a higher appearance variability during training which increases model robustness indicated by a lower performance range across repetitions. This effect could particularly be observed for tumor cell classification for all tumor indications.

When deploying the models to unseen target domains (in our case, new tumor indications), a general drop in performance could be observed. The performance difference between the source and target domain was larger for tumor cell classification than for the non-specified cells. This effect, however, is to be expected as tumor cell morphology is assumed to be more heterogeneous. The non-specified cells are likely composed of vascular cells, immune cells, cancer-associated fibroblasts, and mesenchymal stem cells,^30^ which can be found in the tumor microenvironment of most neoplasms independent of the tumor indication. Surprisingly, the CD3^+^ cell detection also showed large differences in performance across tumor indications even though T-lymphocytes should have a similar appearance across tumors. When looking at the class distributions of the test slide annotations, the TNBC slides showed a considerably lower ratio of CD3^+^ to non-specified cells (0.16) than the other tumor indications (HNSCC: 0.62, NSCLC: 0.45, and GC: 0.37). A single misclassification between these cell types affects the performance metrics of slides with a lower ratio much more severely than slides with a higher ratio. The positive correlation of these ratios with the CD3^+^ classification performance indicates that statistical effects cannot be ruled out. For the differences in tumor cell classification, such a correlation of performance and tumor to non-specified cell ratio could not be observed.

When fine-tuning the models on 1 WSI in the target domains, tumor cell classification performance generally increased and the models became more robust, indicated by a decrease in performance range. These effects could be observed across all target domains and the mean absolute improvement was similar for all tumor indications (see Fig. 8). Overall, fine-tuning could lift the tumor cell classification performance to the same level as training from scratch on the target domains. However, compared to the benchmark models, which were trained with 10 target WSIs, fine-tuning only required the annotation of 1 additional target WSI. Overall, fine-tuning especially benefited models which had been trained with a small number of source-domain images, which can be seen in an increase in performance and robustness. For models trained with a sufficient number of WSIs (>9), this effect was less significant. Nevertheless, fine-tuning seldom impacted the performance negatively, and when it did, the decrease in performance was negligible (see Fig. 8). Taking the low additional annotation expense for fine-tuning the models into account, fine-tuning should generally be considered when transferring a trained model from the source to the target domain.

In some cases, a fine-tuned model was even able to outperform the benchmark model. A model pre-trained with 15 HNSCC WSIs and fine-tuned with 1 target WSI has been presented with a larger variety of cells than the benchmark model trained with 10 target WSIs which could have resulted in this increased performance. Additionally, the performance of the benchmark models significantly varied across tumor indications, especially for the differentiation of tumor and non-specified cells. For NSCLC, tumor cell classification was more difficult for the model to learn than for the other tumor indications. This is likely linked to the highly heterogeneous morphology of NSCLC compared to the other tumor indications included in this work. Another possible explanation for this inferiority could be the presence of (intra-)alveolar macrophages, a cell type specific for lung tissue with highly variable morphology. If present, these cells were annotated as “non-specified” but their morphology can be closer to tumor cells than to other cell types of the tumor stroma (e.g., fibroblasts and endothelial cells).

When comparing the detection results to our human annotators, the agreement was comparable to the inter-annotator agreement for almost all tumor indications. The visual examples, however, highlighted challenges in differentiating tumor and non-specified cells, especially at tumor margins. These regions, however, were also identified as causes for disagreement among our human raters. To further improve the model’s performance, it could be trained with a more consistent ground truth, e.g., a pathologist consensus or additional tumor-specific IHC staining to generate a more reliable “gold-standard”. Alternatively, active learning could be implemented to iteratively improve cell annotations whilst minimizing the additional annotation overhead.

All experiments presented in this study were conducted on procured samples. In a clinical setting, several additional influence factors have to be considered that can potentially significantly influence algorithm robustness. For example, samples can strongly vary in quality, due to tissue deterioration or staining artifacts. Furthermore, previous studies have shown that algorithmic performance can also decrease on samples from different pathology labs or digitized by different slide scanning systems.^10^^,^^31^^,^^32^ We have added preliminary experiments on a small qualitative dataset covering the most common artifacts and tissue morphologies that can challenge the algorithm when deployed in clinical practice. These experiments can be found in Appendix B. Overall, the selected regions posed challenges to the algorithm, which can make a complete WSI analysis more difficult. All of our experiments were conducted on selected fields of interest and the algorithm was therefore never exposed to artifacts or atypical tissue morphologies during training. By including these regions in the training dataset, predictions in these areas could be improved, which could be considered for future work. If the analysis of a complete WSI is of interest, the T-lymphocyte detection algorithm could also be integrated into a more complex image analysis pipeline, where common artifacts are first detected and removed from further analysis, tumor areas are separated from tumor necrosis using a tumor segmentation model, and the T-lymphocyte detection thereby limited only to tumor regions. This cascaded image analysis would allow for the use of task-specific algorithms which are expected to perform better at their designated task than a T-lymphocyte algorithm developed to account for all morphological subtypes and artifacts that can be encountered during WSI analysis.

## Conclusion and outlook

The presented work has evaluated the robustness of a T-lymphocyte detection algorithm under limited data availability and domain shifts introduced by different tumor indications. By leveraging existing software tools, we generated a high number of single-cell annotations in a comparably short time frame, which have shown a high consistency with expert annotations. Furthermore, this semi-automatic annotation pipeline reduces the occurrence of missed cell candidates, which would otherwise require repeated screening of samples or consensus annotations. By using the semi-automatic annotations to train a CNN for the given task, an algorithm was created that is less dependent on manual interaction, e.g., threshold optimization, and can better generalize across sample diversity and different sources of domain shift. Overall, our experiments allow recommendations for the development of T-lymphocyte detection models:

• We recommend using semi-automatic pipelines for collecting single-cell annotation as they enable the generation of a high number of labels with sufficient annotation quality in a comparatively short amount of time. Still, care needs to be taken during this process to curate the semi-automatic results.
• Few CD3^+^ cell annotations (∼500) are sufficient to train a robust model to detect marker-positive cells.
• If the differentiation of tumor or non-specified cells is of interest, e.g., to detect T-lymphocytes that infiltrate the tumor (TILs) or compute cell ratios, a dataset composed of at least 5000 annotations per cell class (average number of annotations on 9 annotated WSIs; for details see Fig. 4) provides a sufficiently high variety of cell morphologies for robust model training.
• When deploying the model to an unseen target domain, we recommend undertaking the annotation effort for at least 1 target slide which can be used to fine-tune the algorithm.
• We recommend always making use of models trained for a similar task in a related domain and employing transfer learning techniques to adapt these models as our fine-tuned models performed on par with the models trained from scratch whilst requiring considerably fewer additional annotations for training.

Future work could focus on alternative domain shifts introduced by different pathology labs, digitization methods, or even different IHC staining agents. These domain shifts could also be approached by using transfer learning or unsupervised methods for domain adaptation, e.g., self-supervised learning or generative models.

## Figures and Tables

**Table A.1:** Detailed overview of dataset with dataset split used for algorithm development and diagnosis according to pathology report. HNSCC: head and neck squamous cell carcinoma, NSCLC: non-small cell lung cancer, TNBC: triple-negative breast cancer, GC: gastric cancer, TNM: tumor-node-metastasis staging.

ID	Dataset			Diagnosis (according to pathology report)	TNM	CD3^+^ cells	
	Train	Valid	Test			[#]	[%]
HNSCC_01	•			Sqamous cell carcinoma	III	16	0.02
HNSCC_02	•			Sqamous cell carcinoma, larynx	IIIA	1010	0.51
HNSCC_03	•			Sqamous cell carcinoma, pharynx	III	54	0.06
HNSCC_04	•			Sqamous cell carcinoma	III	89	0.07
HNSCC_05	•			Sqamous cell carcinoma	II	227	0.25
HNSCC_06	•			Sqamous cell carcinoma, larynx	III	397	0.23
HNSCC_07	•			Sqamous cell carcinoma, pharynx	III	365	0.22
HNSCC_08	•			Sqamous cell carcinoma, pharynx	IV	60	0.06
HNSCC_09	•			Sqamous cell carcinoma, larynx	III	186	0.12
HNSCC_10	•			Sqamous cell carcinoma, larynx	IIIB	190	0.11
HNSCC_11	•			Sqamous cell carcinoma, larynx	I	392	0.22
HNSCC_12	•			Sqamous cell carcinoma, larynx	IIIA	397	0.30
HNSCC_13	•			Sqamous cell carcinoma, larynx	II	125	0.12
HNSCC_14	•			Sqamous cell carcinoma, larynx	II	563	0.44
HNSCC_15	•			Sqamous cell carcinoma, larynx	II	144	0.12
HNSCC_16	•			Sqamous cell carcinoma, tongue		236	0.19
HNSCC_17	•			Sqamous cell carcinoma, tongue		284	0.16
HNSCC_18	•			Sqamous cell carcinoma, oropharynx		76	0.05
HNSCC_19	•			Sqamous cell carcinoma, larynx		251	0.18
HNSCC_20	•			Sqamous cell carcinoma, glottis		291	0.14
HNSCC_21	•			Sqamous cell carcinoma, epiglottis		533	0.22
HNSCC_22	•			Sqamous cell carcinoma, larynx		133	0.10
HNSCC_23		•		Sqamous cell carcinoma, larynx	III	151	0.11
HNSCC_24		•		Sqamous cell carcinoma, tongue	IIIB	91	0.09
HNSCC_25		•		Sqamous cell carcinoma, larynx		217	0.11
HNSCC_26		•		Sqamous cell carcinoma, larynx		149	0.14
HNSCC_27		•		Sqamous cell carcinoma, larynx		497	0.27
HNSCC_28			•	Sqamous cell carcinoma	IV	246	0.25
HNSCC_29			•	Sqamous cell carcinoma, larynx	I	156	0.09
HNSCC_30			•	Sqamous cell carcinoma, larynx	II	529	0.29
HNSCC_31			•	Sqamous cell carcinoma, glottis		581	0.29
HNSCC_32			•	Sqamous cell carcinoma, larynx		392	0.28
NSCLC_01	•			Squamous cell carcinoma, invasive	IV	26	0.02
NSCLC_02	•			Adenocarcinoma, invasive	IV	341	0.25
NSCLC_03	•			Squamous cell carcinoma, invasive	IV	132	0.14
NSCLC_04	•			Squamous cell carcinoma, invasive	IIIB	160	0.07
NSCLC_05	•			Squamous cell carcinoma, invasive	IIIB	197	0.10
NSCLC_06	•			Squamous cell carcinoma, invasive	IIIA	302	0.17
NSCLC_07	•			Squamous cell carcinoma, invasive	IV	96	0.10
NSCLC_08	•			Squamous cell carcinoma, invasive	IV	200	0.15
NSCLC_09	•			Squamous cell carcinoma	IIA	184	0.14
NSCLC_10	•			Adenocarcinoma, micropapillary	IIIA	54	0.04
NSCLC_11		•		Squamous cell carcinoma, invasive	IIIC	275	0.24
NSCLC_12		•		Squamous cell carcinoma, invasive	IIIB	169	0.06
NSCLC_13		•		Squamous cell carcinoma, invasive	IV	181	0.13
NSCLC_14		•		Squamous cell carcinoma, invasive	IIIB	77	0.11
NSCLC_15		•		Adenocarcinoma	IIIA	168	0.09
NSCLC_16			•	Adenocarcinoma, solid predominant	IIIB	870	0.38
NSCLC_17			•	Adenocarcinoma, acinar predominant	IIIB	180	0.17
NSCLC_18			•	Squamous cell carcinoma, invasive	IIIB	106	0.06
NSCLC_19			•	Squamous cell carcinoma, invasive	IIIB	457	0.29
NSCLC_20			•	Squamous cell carcinoma	IIB	133	0.13
TNBC_01	•			Invasive carcinoma	IIIA	177	0.10
TNBC_02	•			Invasive carcinoma	IIIB	521	0.32
TNBC_03	•			Invasive carcinoma	IIIA	172	0.10
TNBC_04	•			Invasive carcinoma	IIIA	191	0.20
TNBC_05	•			Invasive carcinoma	IIIA	97	0.09
TNBC_06	•			Invasive carcinoma	IIIA	145	0.07
TNBC_07	•			Invasive carcinoma	IIIA	324	0.33
TNBC_08	•			Invasive carcinoma	IIIB	37	0.03
TNBC_09	•			Invasive carcinoma	IIIA	149	0.15
TNBC_10	•			Invasive carcinoma	IIIA	258	0.17
TNBC_11		•		Invasive carcinoma	IIIA	734	0.40
TNBC_12		•		Invasive carcinoma	IIIB	155	0.10
TNBC_13		•		Invasive carcinoma	IIIB	384	0.30
TNBC_14		•		Invasive carcinoma	IIIB	638	0.40
TNBC_15		•		Invasive carcinoma	IIIA	276	0.19
TNBC_16			•	Invasive carcinoma	IIIA	126	0.11
TNBC_17			•	Invasive carcinoma	IIIA	61	0.04
TNBC_18			•	Invasive carcinoma	IIIA	293	0.17
TNBC_19			•	Invasive carcinoma	IIIA	383	0.21
TNBC_20			•	Invasive carcinoma	IIIA	115	0.10
GC_01	•			Adenocarcinoma, intestinal-diffuse	IIB	241	0.21
GC_02	•			Adenocarcinoma, intestinal-solid	IIIB	155	0.18
GC_03	•			Adenocarcinoma, intestinal-solid	IIIA	261	0.27
GC_04	•			Adenocarcinoma, intestinal	IIIA	86	0.10
GC_05	•			Adenocarcinoma, intestinal	IIB	226	0.18
GC_06	•			Adenocarcinoma, intestinal	IIA	113	0.11
GC_07	•			Adenocarcinoma, intestinal		190	0.13
GC_08	•			Adenocarcinoma, intestinal		64	0.04
GC_09	•			Adenocarcinoma, intestinal		319	0.17
GC_10	•			Adenocarcinoma, solid		184	0.15
GC_11		•		Adenocarcinoma, intestinal-solid	IIIA	187	0.18
GC_12		•		Adenocarcinoma, diffuse	IIIB	254	0.22
GC_13		•		Adenocarcinoma, intestinal-mucinous	IIA	247	0.17
GC_14		•		Adenocarcinoma, intestinal-solid	IIA	81	0.07
GC_15		•		Adenocarcinoma, intestinal-solid	I	476	0.34
GC_16			•	Adenocarcinoma, intestinal	IIIC	26	0.03
GC_17			•	Adenocarcinoma, intestinal-solid	IIIB	68	0.06
GC_18			•	Adenocarcinoma, intestinal	IIIA	219	0.16
GC_19			•	Adenocarcinoma, intestinal	IIB	107	0.08
GC_20			•	Adenocarcinoma, intestinal	IIB	125	0.10

**Fig. B.1:**
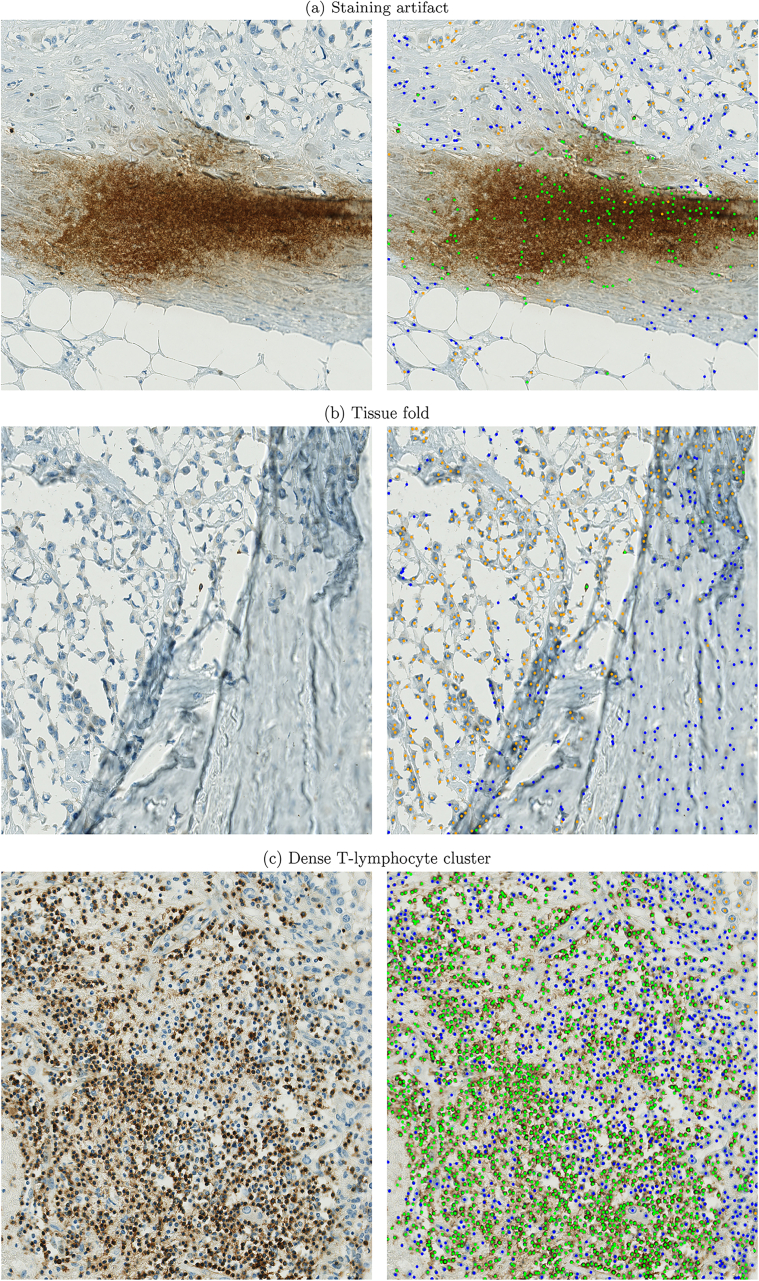

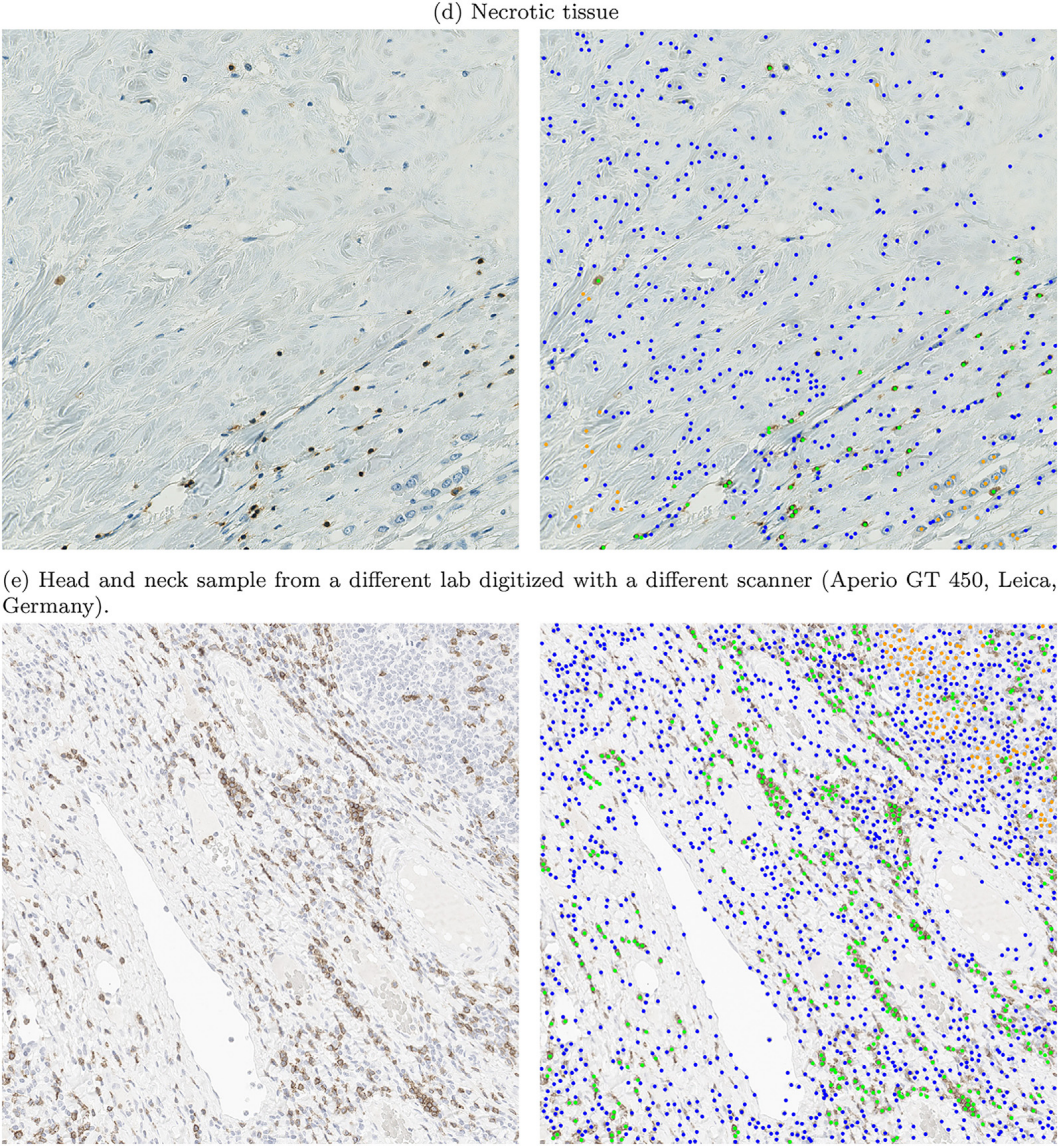
Detection results for common tissue and staining artifacts and morphologically challenging regions (orange: tumor cells, green: CD3^+^ cells, blue: non-specified cells).

## Appendix B Tissue and staining artifacts

The presented workflow has been developed and tested on pre-selected ROIs. These were deliberately placed in areas without tissue or staining artifacts. When deploying the algorithm to a complete WSI, these regions can be challenging and introduce false-positive or false-negative detections. We have conducted preliminary experiments to test the algorithm on these challenging regions and highlight potential weaknesses. Our additional qualitative test set covered 4 common artifacts and morphologies, visualized in Fig. B.1: a staining artifact (Fig. B.1a), a tissue fold (Fig. B.1b), a region with dense clusters of lymphocytes (Fig. B.1c), and a necrotic area (Fig. B.1d). Each of these regions poses its own challenge to the algorithm, often resulting in a high number of false-positive cell detections, e.g., the staining artifact, the tissue fold, and the necrotic region. The dense T-lymphocyte cluster resulted in a lot of correct cell detections but the intense staining produced a light background coloration in some regions, which lead to some false predictions of CD3^+^ cells. Furthermore, dense cell clusters challenge automatic T-lymphocyte detection as strongly overlapping bounding boxes could potentially be removed during NMS. However, the detection results in Fig. B.1c look promising even for dense cell clusters, with few missed cells. For even denser cell clusters, the NMS threshold could be adapted to be less sensitive, however, at the cost of possible duplicate detections. Fig. B.1e shows a CD3-stained head and neck sample that we obtained from a different laboratory. This sample was also digitized with a different scanning system (Aperio GT 450, Leica, Germany), producing WSIs with considerably lower contrast than the Hamamatsu system used for the original dataset of this study. The detection results show that most of the CD3^+^ cells were identified correctly but some tumor cells were mistaken as non-specified cells. Furthermore, some cells (CD3^+^ as well as tumor cells) were not detected at all by the algorithm. When comparing the sample in Fig. B.1e to the samples of the original dataset, the staining intensity appears significantly brighter. This low-contrast appearance likely resulted from a different staining protocol with a lower concentration of staining agent and a lower contrast of the Aperio GT 450 scanner used to digitize the sample. The intensity of the hematoxylin counter-stain might be too low compared to the learned representations and therefore be mistaken as background. Overall, the example shows that the algorithm could in principle be transferred to samples from different labs but fine-tuning techniques would be advisable to overcome the inter-lab domain shift.
